# Niche differentiation of two sympatric species of *Microdochium *colonizing the roots of common reed

**DOI:** 10.1186/1471-2180-11-242

**Published:** 2011-10-27

**Authors:** Michael Ernst, Karin Neubert, Kurt W Mendgen, Stefan GR Wirsel

**Affiliations:** 1 Lehrstuhl Phytopathologie, Fachbereich Biologie, Universität Konstanz, Universitätsstr. 10, D-78457 Konstanz, Germany; 2 Institut für Agrar- und Ernährungswissenschaften, Naturwissenschaftliche Fakultät III, Martin-Luther-Universität Halle-Wittenberg, Betty-Heimann-Str. 3, D-06120 Halle (Saale), Germany; 3 Interdisziplinäres Zentrum für Nutzpflanzenforschung, Martin-Luther-Universität Halle-Wittenberg, Betty-Heimann-Str. 3, D-06120 Halle (Saale), Germany

## Abstract

**Background:**

Fungal endophyte communities are often comprised of many species colonizing the same host. However, little is known about the causes of this diversity. On the one hand, the apparent coexistence of closely related species may be explained by the traditional niche differentiation hypothesis, which suggests that abiotic and/or biotic factors mediate partitioning. For endophytes, such factors are difficult to identify, and are therefore in most cases unknown. On the other hand, there is the neutral hypothesis, which suggests that stochastic factors may explain high species diversity. There is a need to investigate to what extent each of these hypotheses may apply to endophytes.

**Results:**

The niche partitioning of two closely related fungal endophytes, *Microdochium bolleyi *and *M. phragmitis*, colonizing *Phragmites australis*, was investigated. The occurrences of each species were assessed using specific nested-PCR assays for 251 field samples of common reed from Lake Constance, Germany. These analyses revealed niche preferences for both fungi. From three niche factors assessed, i.e. host habitat, host organ and season, host habitat significantly differentiated the two species. *M. bolleyi *preferred dry habitats, whereas *M. phragmitis *prevailed in flooded habitats. In contrast, both species exhibited a significant preference for the same host organ, i.e. roots. Likewise the third factor, season, did not significantly distinguish the two species. Differences in carbon utilization and growth temperature could not conclusively explain the niches. The inclusion of three unrelated species of Ascomycota, which also colonize *P. australis *at the same locations, indicated spatio-temporal niche partitioning between all fungi. None of the species exhibited the same preferences for all three factors, i.e. host habitat, host organ, and time of the season.

**Conclusions:**

The fungal species colonizing common reed investigated in this study seem to exploit niche differences leading to a separation in space and time, which may allow for their coexistence on the same host. A purely neutral model is unlikely to explain the coexistence of closely related endophytes on common reed.

## Background

Traditionally, biodiversity has been explained by the niche partitioning hypothesis, which stresses that coexisting species are differentiated by niche dimensions. On the other hand, the neutral hypothesis proposes that species at the same trophic level colonizing the same space are functionally equivalent [1], because different species have the same likelihood of dispersal, death and birth. Assessment of plant communities has yielded controversial results, some seemed to support the neutral hypothesis [1-3], whereas others did not [4,5]. Attempts have been made to resolve the controversy between the traditional and the neutral hypotheses by integrating stochastic factors into niche-based models [6]. A meta-analysis comparing 158 community studies, covering a wide range of habitats, regions and organisms indicated that the neutral hypothesis explained the observed data well in 8% of the cases [7]. However, 44% of the studies matched traditional niche partitioning models, whereas the remaining studies either matched mixed models or were not assigned. Thus, niche factors appear to be essential in many cases for explaining biodiversity but the integration of stochastic elements may improve interpretation. Most research addressing these hypotheses has been performed with plants and animals. For fungi, such research has focused on arbuscular mycorrhizal (AM) fungi, which are widespread root symbionts of a vast range of plant species. AM fungi promote host nutrition, diversity and survival under biotic and abiotic stress conditions [8,9]. Besides AM fungi, other types of fungal mutualists, for example endophytes, can improve the health and the performance of plants. Studies on endophytes have assessed their occurrences and their influences on their hosts and on plant community structure [10-13]. However, further research is required to elucidate the causes and mechanisms leading to the observed diversity of endophytes.

Common reed (*Phragmites australis *(Cav.) Trin. ex Steudel) has been used as a model to investigate the interactions of a plant with its associated mycoflora and the interactions between different fungi colonizing the same host. Previously, it was found that many different fungi colonized healthy common reed growing in the native freshwater habitats of Lake Constance in the northern alpine forelands of Germany. The number of fungal species identified by cultivation-independent, molecular approaches [14,15] clearly exceeded those isolated by classical cultivation [16,17]. However, only a fraction of the many fungal species present reached a high prevalence, suggesting that competition and niche differentiation may shape these communities.

Abiotic and biotic factors, which distinguish various niches and which may allow some fungal species to dominate over others, are manifold. One approach to identify such factors is to analyze distribution patterns of fungal species observed in classical cultivation schemes, in gene libraries from cloned environmental DNA or in datasets generated using other molecular approaches. The need for sufficient replications in such studies can be met by employing nested-PCR assays that monitor specific fungal species in large collections of field samples. For common reed in Lake Constance these analyses revealed that habitat type and host organ influenced the occurrences of two uncultured fungi [15]. Additional abiotic and biotic factors that may lead to niche differentiation like temperature, pH, carbon, nitrogen, and other resources can be analyzed, if cultured strains are available.

Isolates belonging to the genus *Microdochium *(Ascomycota, Pezizomycotina, Sordariomycetes, Xylariales), were the most frequent among those recovered from *P. australis *under conditions favoring the isolation of endophytes [16]. These *Microdochium *isolates were preliminarily assigned to *Microdochium *sp. and *M. phragmitis*. The former, which was later characterized as *M. bolleyi*, was shown to colonize living roots of reed without causing symptoms [18]. *M. bolleyi *has a broader host range, since it occurs as a minor root pathogen or an endophyte on other grasses as well [19-21]. *M. phragmitis *seems, however, to associate only with reed.

To investigate coexistence, several approaches were used to search for evidence of niche partitioning between fungal species sympatrically colonizing common reed at Lake Constance. Presence-absence patterns were obtained using specific nested-PCR assays on a large set of field samples determining co-occurrences of the two *Microdochium *species and three additional, unrelated species. Furthermore, whether divergent growth temperature optima and resource partitioning could define the niches of the two closely related fungal species was examined.

## Methods

### Cultivation of fungi

The fungal isolates used in this study (Additional file 1) originated from a previously published study [16]. Reference strains were purchased from CBS (Utrecht, Netherlands). All fungi were cultured on 2% malt agar (Biomalt, Villa Natura Gesundprodukte GmbH, Kirn, Germany) at 20°C in the dark.

Mycelial growth rates were determined using three culture replicates for each isolate and each temperature assayed. These ranged from 0°C to 30°C at intervals of 5°C. The mycelial radii for all cultures were determined after 14 d and additionally at 7 d for cultures incubated at temperatures ranging from 15°C to 30°C. Four individual isolates were analyzed for the 5/97-16 sequence type and five isolates for the 5/97-54 sequence type. Two reference strains were used for *M. bolleyi *(CBS 137.64, CBS 172.63), and for *M. nivale *(CBS 110.94, CBS 320.78), respectively. Where applicable, data from strain replicates were combined and averaged. The data were analyzed statistically using the Dunnett test and multifactorial analysis of variance (MANOVA) that separately analyzed the growth rates of the isolates belonging to a species and their individual replicates (confidence limits at *P *< 0.05). Both tests were implemented using JMP software version 4.04 (SAS Institute, Cary, NC, USA).

### DNA extraction, PCR, sequencing and phylogenetic analysis

DNA preparations from fungal mycelia were performed as described previously [22]. DNA preparations from reed tissues used for nested-PCR assays had been conducted earlier [17,22] and were kept frozen at -20°C. Reed was harvested from Lake Constance (Germany) at four sites, described previously [16].

DNA sequences of the ITS (internal transcribed spacers) rDNA region from fungal isolates were produced, assembled, aligned and edited as previously described [22].

**Figure 1:**
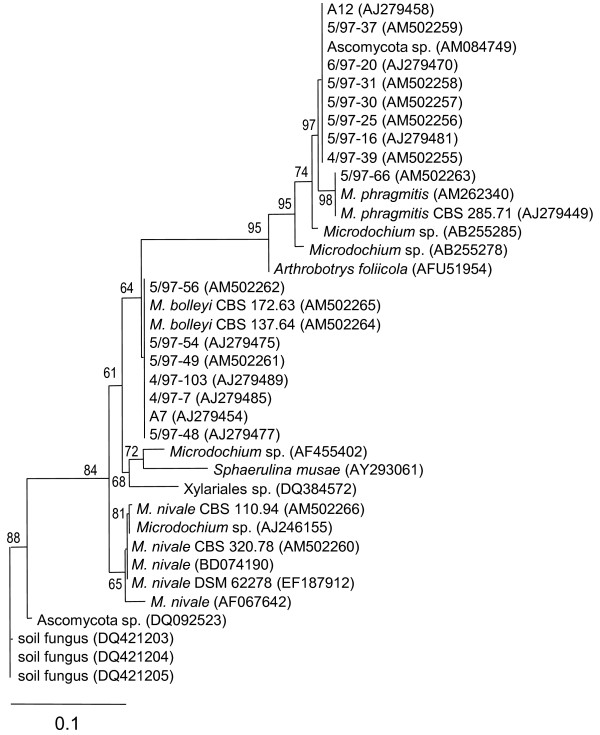
**Molecular phylogeny of *Microdochium *spp**. Molecular phylogeny obtained using Maximum Likelihood analysis on ITS rDNA, displaying the relationships between 37 sequences originating from reed isolates, their closest database matches, as well as additional references. Accession numbers are provided in brackets. Reference sequences are shown as annotated in the source database. Support of branches is shown when higher than 50%.

Phylogenetic analysis relied on the alignment of 37 sequences created using the software ClustalX ftp://ftp.ebi.ac.uk/pub/software/mac/clustalx and then manually adjusted. The alignment comprised the ITS1-box, the 5.8S rRNA gene, and the ITS2-box. Besides the sequences of isolates listed in Additional file 1 sequences from related fungi were included that were detected using BlastN searches in public databases. The program PAUP Version 4.0b10 was used to generate the phylogenetic tree depicted in Figure 1[23]. The BioNJ method with the HKY85 setting for distance measures was used to create a tree that served to estimate the proportions of invariable sites and gamma shapes. A heuristic search under the maximum likelihood criterion and the GTR+G+I substitution model, using the neighbor-joining tree as input, was created. The confidence of the resulting ML tree was estimated using 1000 quartet puzzle steps.

Sequences obtained during this study were deposited in the EMBL-EBI Nucleotide Sequence Database (European Molecular Biology Laboratory-European Bioinformatics Institute; http://www.ebi.ac.uk/) under the accession numbers AM502255 to AM502266 (Additional file 1).

### Nested-PCR assays

DNA preparations originated from 251 samples of 66 standing reed plants that were harvested from Lake Constance from July 1998 to August 2001 [17]. The same DNA preparations had been used earlier to determine the distribution of three additional fungi that were frequently observed in common reed using specific nested-PCR assays [15,17]. These previous data allowed assessment of fungal co-occurrence at a broader scale investigating whether other fungi may have influenced the prevalence of *Microdochium *spp. Two of the additional fungi were uncultured Ascomycota and were originally identified using a molecular approach [15]. They were designated as Ms7Mb4 (related to *Podospora*) and Ms43Mb21 (distantly related to an uncharacterized ericoid mycorrhizal fungus). The third fungus was an endophyte, *Stagonospora *sp. 4/99-1 that originated from cultivation [17].

The first PCR-step of the two-step nested-PCR assay targeted the Eumycota using the primers ITS1F and ITS4. Reaction mixtures contained: 100 ng of DNA, 2 mM MgCl_2_, 0.2 mM dNTPs, 0.5 mg/mL bovine serum albumin, 0.25 μM of each primer and 0.05 U/μL of recombinant Taq DNA Polymerase (MBI Fermentas) in a total volume of 25 μL. An initial denaturation step at 94°C for 150 s was followed by 40 cycles of the following protocol: 94°C for 30 s, 55°C for 15 s and 72°C for 45 s plus one additional second per cycle. The reaction was terminated by a final extension at 72°C for 10 min. The second PCR step applied specific primers annealing at the highly variable ITS1 and ITS2 boxes. These primers were: 5/97-54/ITS.F2 (5'-GGT GCT GGA AAC AGT GCT GCC AC-3') and 5/97-54/ITS.R2 (5'-GTC GTC TGG CCG GCT TGC AG-3') that were derived from the sequence of isolate *M. bolleyi *5/97-54 (Accession no. AJ279475), and 5/97-16/ITS.F2 (5'-ACC CGA AAG GGT GCT GGA AG-3') and 5/97-16/ITS.R2 (5'-TTG GCT ATC GTC TAG ACG TGT TCA A-3') that were derived from the sequence of *M. phragmitis *5/97-16 (Accession No. AJ279481). Reaction mixtures contained: 0.25 μL of the first PCR reaction, 1.5 mM MgCl_2_, 0.2 mM dNTPs, 0.5 mg/mL bovine serum albumin, 0.125 μM of each primer and 0.05 U/μL of recombinant Taq DNA Polymerase in a total volume of 25 μL. Reactions with primers 5/97-54/ITS.F2 and 5/97-54/ITS.R2 included an initial denaturation step of 94°C for 120 s that was followed by 5 cycles of a touch-down protocol (94°C for 30 s, 82°C for 45 s with a decrease of 1°C per cycle) and then by 40 additional cycles (94°C for 30 s, 77°C for 45 s plus one additional second per cycle). This was followed by a final extension at 77°C for 10 min. Reactions with primers 5/97-16/ITS.F2 and 5/97-16/ITS.R2, basically followed the same scheme but had an initial annealing temperature of 77°C at the first cycle, followed by a touch-down to 72°C. Positive and negative controls included genomic DNAs of target and non-target fungi, respectively.

Results of nested-PCR assays were scored as 0 vs. 1 and statistically analyzed using a contingency table and a binomial distribution test (*P *< 0.05) with the Bonferroni correction. The co-occurrences of two fungi in the same samples were examined using pair-wise contingency analysis and two-sided Fisher's Exact test (confidence limits at *P <*0.05) to determine deviation from a random distribution, either positive or negative. Fisher's Exact test provides a precise likelihood for the observed distribution, but is restricted to pair-wise analysis. These statistical analyses were performed using JMP version 4.04. Analyses of co-occurrences of several species were carried out with the Co-occurrence module in the software EcoSim Version 7.72 http://garyentsminger.com/ecosim/index.htm. EcoSim applies a Monte Carlo approach to create a random distribution of data for statistical testing that is compared to the experimental data to test the null hypothesis that the co-occurrence patterns observed in the field samples result from random variation (confidence limits at *P <*0.05) [24]. The recommended default settings were used except for the number of randomized data matrices generated by the software, which was increased to 10000. It had previously been suggested that deviation from other default program settings, that keep the number of species observed in each sample ("fixed columns") constant, as well as the sum of the incidences of each species ("fixed rows") for the randomizations, could result in misleading assertions [25].

Canonical correspondence analysis (CCA) with PC-ORD version 5.10 (MjM Software Design, Gleneden Beach, OR) was used to assess the degree to which each of three factors analyzed contributed to the frequencies of the fungi detected in the field samples. CCA used the incidences of five species examined for 46 distinct habitat-organ-month combinations. In CCA setup, axis scores were standardized using Hill's method. Axes were scaled to combine representation of species and stands. Stand scores were treated as linear combinations of factors. Graph ordination was set up in two dimensions to present the two most important factors. A Monte Carlo permutation test based on 999 random permutations was applied to test the null hypothesis that the community was independent of the analyzed factors.

### Registration of substrate utilization spectra

*Microdochium *isolates were grown in liquid medium containing 4 g/L glucose, 10 g/L malt extract, 4 g/L yeast extract [26] for 3-7 d at 20°C and 120 rpm to obtain the inoculum for the physiological tests. The reed strains A7, 4/97-7, 5/97-48, 5/97-49, and 5/97-54 were taken for *M. bolleyi*, whereas 4/97-39, 5/97-16, 5/97-30, and 6/97-20 represented *M. phragmitis*. In addition, reference strains from CBS, (Additional file 1) were analyzed. Mycelia were harvested using filtration, washed with autoclaved distilled water and re-suspended in 2% carrageenan type II (Sigma, Deisenhofen, Germany) to provide an OD590 of 0.05. Each well of a BIOLOG SF-N2 plate (Merlin Diagnostika GmbH, Bornheim, Germany) was inoculated with 100 μl of mycelial suspension. These microtiter plates contained 95 different carbon sources and one control well without any carbon source. Plates were incubated for 10 d at 21°C in the dark. Thereafter, absorption at 560 nm was recorded using an ELISA reader (SLT Spectra, SLT Laborinstrumente GmbH, Grödig, Austria). After exporting the data to Microsoft Excel, the absorption in the control well was defined as 0% growth and that of the well with the maximum absorption as 100%. All other values were scaled in relation to these limits. For each isolate tested, three independent experiments were performed and the transformed results were averaged. The t-test and the Dunnett test in JMP were used to assess the variation between species for each carbon source using the average values of each isolate (confidence limits at *P <*0.05). Furthermore, the overall similarity of carbon usage patterns between species was compared using the Niche Overlap module in EcoSim [24]. All four EcoSim randomization algorithms (RA1-RA4) were used to generate 10000 simulated data matrices in each case. For all other parameter settings, the default was used.

## Results

### Molecular characterization of *Microdochium *isolates

A molecular phylogeny of the ITS region was generated that included previous sequences from *Microdochium *spp. [16], new sequences from Lake Constance reed isolates and from CBS reference strains (Additional file 1), and in addition, sequences from databases that were identified by BlastN searches. The phylogeny indicated the existence of three different sequence variants among the isolates colonizing common reed at Lake Constance (Figure 1). Eight isolates had identical sequences and were typified by the previously described isolate 5/97-16 [16]. This sequence variant had 98.4% identity to the reference *M. phragmitis *(CBS 285.71). A single isolate, 5/97-66, was identical to CBS 285.71. We treated all these isolates as *M. phragmitis*. This degree of similarity was clearly higher than the limit of 97% that had previously been suggested to differentiate fungal species using their ITS sequence [27,28]. Furthermore, because intraspecific variation in the rRNA gene cluster is known in eukaryotes including fungi, a higher threshold value may introduce the risk of wrongly dividing isolates belonging to a single species into different species. A previous study found that intraspecific ITS variation ranged from 0.16 to 2.85% in Ascomycota and Basidiomycota [29]. Another group of seven isolates had sequences that formed a cluster with the references *M. bolleyi *CBS 137.64 and CBS 172.63. They diverged by at most 0.5% from each other. Therefore, and because typical morphological characters were highly similar compared to these references (data not shown), the previously described *Microdochium *sp. typified by isolate 5/97-54 [16] was treated here as *M. bolleyi*. None of the isolates from reed clustered with references belonging to *M. nivale *or any of the other species included in the phylogram.

### Nested-PCR assays indicate niche differentiation of *Microdochium *spp

To examine whether colonization of *P. australis *by the two species of *Microdochium *reflected stochastic patterns or niche differentiation two nested-PCR assays were designed that specifically targeted the ITS sequence of the 5/97-16 and of the 5/97-54 sequence variants. The specificities of these assays were tested using genomic DNA preparations as templates that were extracted from the fungal isolates typifying the *Microdochium *sequence variants identified above and from additional isolates belonging to other genera of Ascomycota that had been recovered from *P. australis *earlier [16]. Genomic DNA from aseptically grown *P. australis *served as an additional negative control. As anticipated, the first PCR step, which used standard primers targeting the Eumycota, yielded reaction products with all fungal templates (Additional file 2A). The second PCR steps using primers directed against the individual *Microdochium *species yielded reaction products only with DNA from the targeted fungi (Additional file 2B-C).

The incidences of the two *Microdochium *species in 251 DNA samples covering a period of three years, four host organs, i.e. rhizome, root, stem, and leaf, and two contrasting habitat types, i.e. flooded and dry, were analyzed. Both targets were generally detectable in all organs, at all sites and throughout the seasons. The overall detection frequency was 22% for *M. phragmitis *and 27% for *M. bolleyi*. From each nested-PCR series one randomly chosen product for each host organ was sequenced to validate the correctness of the method employed. Each of the eight PCR-products corresponded to the respective previously determined sequences (data not shown).

**Figure 2:**
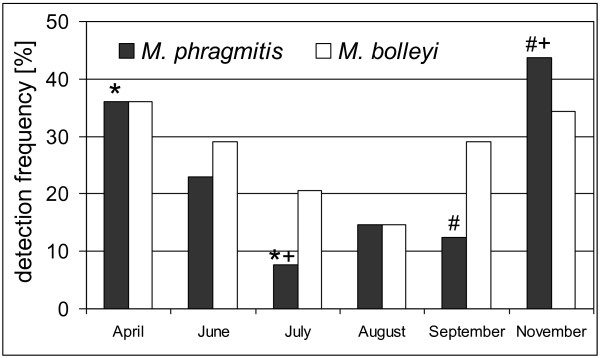
**Seasonal variation of *Microdochium *spp. on Lake Constance reeds**. Summary of nested-PCR assays on 251 DNA preparations from tissue samples of *P. australis *harvested over a period of three years. Detection frequency for each target shows the percentage of samples producing a band after the second step of the nested-PCR. Results from all sites and all host organs were pooled. Symbols on top of the columns indicate significant variation between the respective months when analyzing each fungus separately (binomial test with *P <*0.05). Occurrences of *M. phragmitis *differed significantly when comparing April with July (*), July with November (+), and September with November (#).

In general, the results from the nested-PCRs on the field samples indicated for both targets, but especially for *M. phragmitis*, a reduced prevalence during the warm summer months when the data were pooled across host habitat and host organ (Figure 2). Statistical support for this observation was obtained for *M. phragmitis *when comparing its minimum, i.e. July, in a pair-wise manner with the other months that demonstrated a significant difference to April (binomial test, *P *= 0.006) and November (*P *= 0.007). In addition, the variance between September and November was also significant (*P *= 0.007). When applying the stringent Bonferroni corrections on an analysis testing all months against each other, all variations appeared non-significant. Variations in the corresponding data for the other target, *M. bolleyi*, did not show any significance, neither when analyzed in a pair-wise manner nor in a total analysis. For both targets, there was no statistical support for seasonal variation when evaluating the results for the individual host organs separately (data not shown, binomial test with *P <*0.05, Bonferroni corrected). When comparing the detection frequencies of the two fungi against each other none of the apparent variations proved to be significant for any month when the data were pooled across organs (binomial test with *P <*0.05, Bonferroni corrected) (Figure 2).

**Figure 3:**
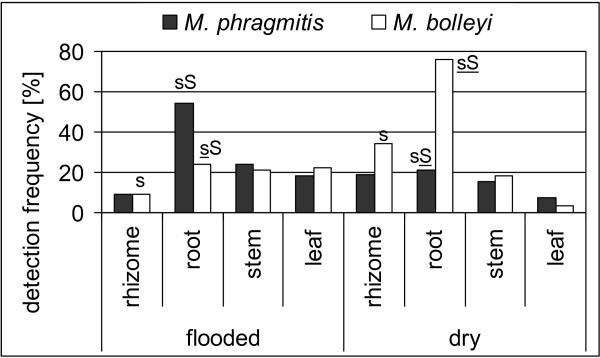
**Habitat preferences of *Microdochium *spp. on Lake Constance reeds**. Summary of nested-PCR assays on 251 DNA preparations from tissue samples of *P. australis*. Detection frequency for each target shows the percentage of samples producing a band after the second step of the nested-PCR. Results from all seasons were pooled. Small letters compare variation between the two habitat types when analyzing each target species and each host organ separately (binomial test with *P <*0.05). Capital letters compare variation between the two species when analyzing each host organ and each habitat separately (binomial test with *P <*0.05). S/s, variation is significant; non-significant variation is not indicated. Underlined letters indicate that the variation remains significant after Bonferroni correction.

Statistical analysis of variation with respect to the colonized host organ revealed for both, *M. phragmitis *and *M. bolleyi*, a significant preference for roots (binomial test with *P <*0.05, Bonferroni corrected). Besides host organ, also the host habitat affected the incidences of the fungi. *M. phragmitis *occurred significantly more frequently at flooded sites compared to dry sites (27% vs. 16%, binomial test, *P *= 0.0385) when the data were pooled across organ. The opposite result was obtained for *M. bolleyi *(19% vs. 34%, binomial test, *P *= 0.0110). When examining variation resolved for all host organ-habitat type combinations (Figure 3, small letters), *M. phragmitis *showed a significant preference for roots at flooded sites (*P *= 0.0127), whereas *M. bolleyi *significantly preferred roots (*P *= 0.0002) and in addition rhizomes (*P *= 0.0386) at the dry sites. Comparisons between the two species showed that roots were the only organs with significantly contrasting preferences for the habitat type (root-flooded: *P *= 0.0213; root-dry: *P *= 0.00004) (Figure 3, capital letters).

### Carbon utilization patterns of *Microdochium *spp

To determine whether resource partitioning, as a biotic attribute, may have contributed to these findings the potential of *Microdochium *spp. to utilize 95 different carbon sources was tested *in vitro*. The EcoSim Niche Overlap module was used to evaluate the overall similarity in carbon usage. The niche overlap index in the experimentally obtained data set was 0.9733, whereas the mean of the simulated matrices was 0.7127, using default parameters for calculation (RA3 model). This difference was statistically significant (*P <*0.05), and thus indicated that the carbon usage of the two species was overall more similar than expected by chance. The application of alternative parameters for the calculation (i.e. the RA1, RA2, and RA4 models) led to the same conclusion. In addition, intra-species comparison of different strains belonging to the same species showed that within each of the two species there were significantly more resource overlaps than expected by chance (data not shown).

Although the carbon utilization capabilities of the two species were similar, specific differences existed, which were statistically assessed using t-tests. Significant differences between the two species (*P <*0.05) were observed for 21 substrates (22.1%) (Additional file 3). In addition, the application of the Dunnett test rendered essentially the same results (not shown). *M. bolleyi *grew significantly better than *M. phragmitis *on 10 of the 95 carbon sources tested (Additional file 3). Conversely, *M. phragmitis *grew significantly better than *M. bolleyi *on 11 carbon sources (Additional file 3).

### Temperature ranges for growth of *Microdochium *spp

**Figure 4:**
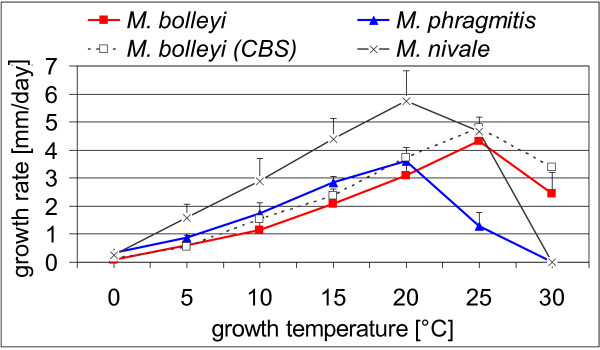
**Growth rates of *Microdochium *spp**. Three replicated assays were recorded for each isolate and each temperature. For each taxon up to five independent isolates were tested. Results show total averages from all replicated assays and all isolates. The tested reed isolates were for *M. phragmitis *4/97-39, 5/97-16, 5/97-30, and 6/97-20, and for *M. bolleyi *A7, 4/97-7, 5/97-48, 5/97-49, and 5/97-54. The tested reference strains were for *M. bolleyi *CBS 172.63 and CBS 137.64, and for *M. nivale *CBS 320.78 and CBS 110.94. Bars indicate standard deviations.

The potential effect of temperature, as an abiotic attribute, was tested to determine if it could distinguish these fungi and explain their observed distributions in field samples. All reed isolates of each species exhibited highly similar growth rates at all temperatures tested. *M. bolleyi *isolates had a maximum at 25°C and were still able to grow at 30°C (Figure 4). *M. phragmitis *isolates had growth rates a little higher than those of *M. bolleyi *up to 20°C. However, they grew much more slowly above 20°C, which was their maximum, and they completely ceased to grow at 30°C. This also applied to *M. nivale*, which was used as an outgroup. When such cultures were transferred to a temperature of 15°C the colonies started to expand again, indicating that these fungi were still viable. Dunnett tests (*P *< 0.05) on the averaged growth rates of the *M. phragmitis *and *M. bolleyi *isolates indicated significant differences for all temperatures tested except 20°C. Multifactorial analysis of variance (MANOVA, *P *< 0.05) yielded the same result.

Whether temperature optima determined *in vitro *could correspond to soil temperature at sites where the fungi were originally isolated was investigated. Three data loggers were buried at a soil depth of 20 cm at one of the original locations on the shores of Lake Constance to record hourly ambient temperatures from March 2005 to February 2006 (K. Neubert & J. Nechwatal, data not shown). The average annual soil temperature was 11.1°C with a maximum reaching slightly above 21°C, which occurred for only a few days in summer. Both species grew equally well *in vitro *at 21°C.

### Co-occurrences of five fungal species

Four statistical tests were used to analyze the occurrences of five fungi, i.e. the binomial distribution test, the EcoSim Co-occurrence module [24], Fisher's Exact test and Canonical Correspondence Analysis (CCA). Except for CCA, one combined data set, including nested-PCR results from all 251 samples, was analyzed simultaneously using each method, and in addition, several partial data sets were analyzed to examine occurrences for season, host organ, host habitat, and for the combination of organ plus habitat, respectively.

**Figure 5:**
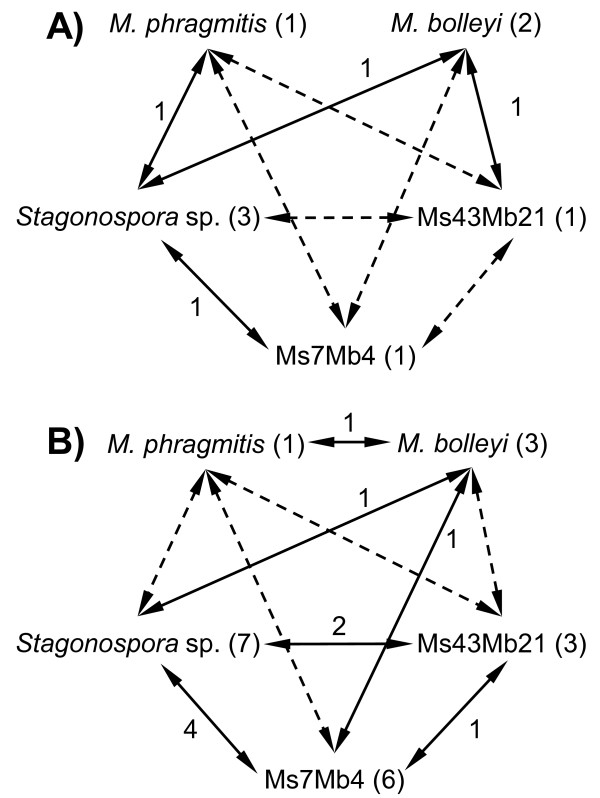
**Niche differentiations of five fungal species with respect to time and space**. Summary of nested-PCR assays on 251 DNA preparations from tissue samples of *P. australis*. Pair-wise species comparisons were conducted using binomial tests with *P <*0.05. Straight arrows indicate variations that remained significant after Bonferroni corrections, broken arrows variations that were additionally significant when Bonferroni corrections were omitted. Numbers at the arrows give the incidences of significant results for a species pair and those in brackets for a given species, respectively. Numbers refer to Bonferroni-corrected comparisons. A) Seasonal variation by months; B) Spatial variation by host organ plus habitat-type.

First, the binomial distribution test (*P <*0.05) with Bonferroni corrections was applied to examine whether within a given data set the total incidence of one species differed significantly from that of another species. Three of ten species pair comparisons using the undivided data set showed significantly contrasting occurrences (Additional file 4). These three comparisons involved Ms43Mb21, which was generally less prevalent than all other species. Although season did not significantly separate the species pair *M. phragmitis *- *M. bolleyi *(as mentioned above), the inclusion of the three additional species showed that this factor contributed to the separation of the five species. Four of 60 species pair comparisons (6.7%) using data sets divided by months (ten species pairs, six months) showed significant differences (Figure 5A, Additional file 4). Nine of 40 species pair comparisons (22.5%) using data sets divided by host organ showed significant differences (Additional file 4). Five of 20 species pair comparisons (25%) using data sets divided by habitat type showed significant differences (Additional file 4). Ten of 80 species pair comparisons (12.5%) using data sets divided by the combination of organ plus habitat showed significant differences (Figure 5B, Additional file 4).

The second statistical test was the Co-occurrence module of EcoSim. In a total data set comprising all five species, significantly less co-occurrence was observed compared to the null hypothesis (*P <*0.05; data not shown). The analyses of data matrices that reflected the distributions of the five species in the individual months exhibited significantly decreased co-occurrences in August and September. Accordingly, assessment of individual organs demonstrated significantly decreased co-occurrences for stem. Both habitats surveyed, dry, and flooded, showed significantly decreased co-occurrences. From the eight organ-habitat combinations, only stems from the dry habitat exhibited a significant decrease. We did not observe a significant increase of co-occurrence in any of the analyses.

The third statistical test applied was Fisher's Exact test (*P <*0.05) with Bonferroni corrections to determine if certain species pairs may co-occur significantly more or less frequently in the same samples than expected by chance. Three of ten species pair comparisons (*M. bolleyi *vs. Ms7Mb4 and vs. Ms43Mb21, respectively, and Ms7Mb4 vs. Ms43Mb21) using the undivided data set showed significantly more co-occurrences (Additional file 5). Only the pairing of *Stagonospora *sp. vs. Ms7Mb4 co-occurred less frequently than expected by chance. Neither the analyses of the 60 species pair comparisons using data sets divided by months nor those of the 40 comparisons using data sets divided by host organ showed any significant differences when applying Bonferroni corrections (Additional file 5). In contrast, examination of data sets separated for host habitat revealed that *M. bolleyi *co-occurred with Ms7Mb4 and Ms43Mb21 more frequently at the dry habitat than expected by chance. Under the same conditions, *M. bolleyi *co-occurred with *Stagonospora *sp. less frequently. None of the 80 pair-wise species comparisons that examined data sets divided by the combination of organ plus habitat showed significantly increased or decreased co-occurrences (Additional file 5).

**Figure 6:**
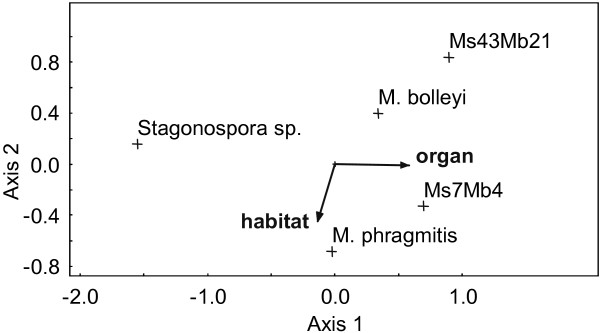
**Canonical correspondence analysis**. CCA biplot ordination for the effects of space defined by plant organ and habitat type assessing five fungal species on reeds at Lake Constance. Axes 1 and 2 explain 32.9% and 5.5% of the variation, respectively. Monte Carlo permutation test on axis 1: *P *= 0.0010.

Finally, CCA was used to estimate which of the analyzed factors most influenced the occurrences of five species in all samples analyzed. Space at the level of organ explained 32.9% of the observed total variation, whereas space at the level of habitat and time at the level of months did so for 5.5% and 0.1%, respectively. A plot including the two main axes indicated that all five species were well separated for at least one factor (Figure 6). It underlined that *Stagonospora *sp. was distinguished from the other species mainly because of its distinct organ preferences. The CCA plot confirmed that habitat type was most important for separation of the two *Microdochium *species. For the remaining species, both organ and habitat determined their separation.

## Discussion

Previous studies have indicated that fungal endophytes may coexist at very small scales. In this study, niche partitioning between two endophytic species of *Microdochium *sympatrically colonizing *Phragmitis australis *was assessed. *M. bolleyi *and *M. phragmitis *were found to be significantly segregated for host habitat, but not for host organ and season. However, when additional, unrelated fungi that colonize the same host were also included in the analyses, the latter two factors were also found to contribute to niche partitioning.

Several factors can cause niche differentiation between endophytes, which may attenuate competition and thus allow for a high fungal diversity on the same host species. One factor is space, which is with respect to endophytes hierarchically structured from continent to region, to habitat, to host individual, to host organ, and further down to the level of host cells. Two of these levels, i.e. the habitat type and the host organ, were analyzed. Both, *M. bolleyi *and *M. phragmitis*, preferentially colonize the same organ, i.e. roots, confirming an earlier result [16]. Within the limits of detection, nested-PCR assays in this study indicated that *M. bolleyi *occurs more frequently on roots at dry sites, whereas *M. phragmitis *occurs more frequently on roots at flooded sites. This suggests spatial niche partitioning at the level of habitat type. Like all molecular assays employing fungal genomic DNA extracted from field samples, the assays from this study cannot distinguish between growing and dormant cells, and thus cannot provide details on metabolic activities or developmental stages. In addition, a possible introduction of bias against rare templates during the first stage of the nested-PCR has to be considered, which would produce false-negative results in case of fungi present at very low abundance. However, if the first step of nested-PCR comprises as many cycles as used here rare templates will be over- not under-amplified, as previously shown [30]. Thus, for assessment of presence-absence data nested-PCR is a highly specific and sensitive method.

Further support for an influence of spatial niche partitioning on the composition of the reed-associated fungal community was obtained when occurrences of three additional species were also considered. Both binomial tests and CCA indicated that all five species were differentiated by host organ and / or habitat. Since *P. australis *has a vast geographical distribution, it would be interesting to assess the factor space in structuring fungal communities at higher hierarchical levels in the future.

The importance of space in affecting fungal community composition has previously been acknowledged. Much of this information comes from pathogens of agronomically important crops [31] and from mycorrhizal fungi [14,32-36]. In addition, endophyte communities seem also to be influenced by the factor space [37-39]. However, in contrast to other types of fungi, little is known about the causes leading to spatial differentiation in endophytes. At the same sites examined in this study an even more distinct preference for the habitat type was previously noted for AM fungi that were not observed at flooded sites at all, whereas at the dry sites, 21 phylotypes were detected at various frequencies [14]. Vertical distribution patterns of reed-associated fungi have been recorded in a brackish tidal marsh, with diverse communities depending on the leaf layer [40]. Site-dependent differences in reed stands are known for Oomycota, where some species preferred either dry or flooded sites [41]. It seems likely that it is not space *per se*, but rather specific physico-chemical features of the respective sites that cause such differences.

Another factor that can cause niche differentiation between fungal endophytes is time, resolved here at the scale of individual months of the season. The progress of the season drives host developmental processes like the emergence of shoots and leaves in spring and senescence in autumn, and thus dynamically modifies the niches available to plant-associated fungi. The occurrences of *M. bolleyi *and *M. phragmitis *were similar for season. Thus, seasonal niche partitioning does not seem to significantly separate *Microdochium *spp. on common reed. However, binomial tests indicated that time was a factor involved in the separation of the five species analyzed.

The contribution of seasonal variation to fungal community variation has previously been recognized. Endophytic colonization of tropical cacao trees increased with leaf age and partially protected the host against pathogenic *Phytophthora *sp. [42]. Similarly, endophytic diversity increased during leaf development in *Camellia japonica*, whereas epiphytic diversity remained stable with season [43]. Seasonal succession was also demonstrated for the mycoflora in a Colorado mountain soil that changed substantially between spring and summer, suggesting functional differentiation [44]. Seasonal variation has been found in an aquatic fungal community decomposing plant debris in streams [45]. In reed stands at Lake Constance, Oomycota populations were shown previously to exhibit seasonal variation [46]. For the reed pathogen *Pythium phragmitis*, minimal detection in August resembled the decrease of *Microdochium *spp. during the summer.

Temporal niche differentiation thus contributes to the separation of the five species examined, although to a lesser extent than space. Thus, niche differences resulting from abiotic or biotic attributes seem to separate these fungi and may explain their coexistence on the same host. Temperature was one attribute that distinguished the two *Microdochium *species *in vitro*. *M. phragmitis*, which occurs more frequently at flooded sites, grows faster at lower temperatures, whereas *M. bolleyi*, which prefers dry sites, grows faster at higher temperatures. For most of the year, based on the *in vitro *growth rates, temperatures existed in the soil at which *M. phragmitis *would grow faster than *M. bolleyi *if additional factors such as competing fungi are not considered. In this context, temperature contributes to the differentiation of other *Microdochium *species [47,48].

Other attributes may be involved in spatial niche differentiation for habitat type observed for *Microdochium *spp. Carbon usage patterns of the two species were found to overlap significantly more than expected by chance, although certain substrates, including compounds of the central carbon metabolism, secondary sugars, and sugar alcohols, are utilized differentially. In *P. australis *site-dependent variations for central metabolites were reported [49]. Basal culm internodes from flooded sites had higher total amino acid and lower total carbohydrate contents than those from dry sites. Several metabolites were individually recorded in that study, but none of those varying for habitat type could explain the contrasting habitat preferences of the two *Microdochium *species when considering the results of the BIOLOG experiments.

Earlier studies have noted that host-derived carbohydrates might affect the occurrences of plant-associated fungi. Root-associated endophytes grew better *in vitro *on low concentrations of certain carbohydrates than rhizosphere and soil fungi retrieved from the same Austrian grassland [50]. It was suggested, "that plant sugars or sugar alcohols may constitute signals that facilitate adaptation of certain fungi to a specific host plant". Some of such compounds are differentially utilizable by *Microdochium *spp. Another study reported that *Neotyphodium *endophytes were inhibited *in vitro *by high concentrations of hexose and were incapable of utilizing xylose and arabinose [51]. These findings were supported by results showing that *Neotyphodium lolii *grows more slowly in varieties of its host *Lolium perenne *bred for intrinsically high sugar concentrations [52]. For AM fungi, it was suggested that competition for the same carbon sources present in the same niche caused differential colonization [53]. A report comparing ericoid and orchid mycorrhizal fungi found that carbon source utilization was generally quite similar *in vitro *except for distinct differences for tannic acid and certain amino acids [54]. These publications indicate that the quality and the quantity of carbon sources available in the host may be one of the attributes influencing the composition of the associated fungal community.

Although the BIOLOG system provides interesting insights in the capacity of fungi to utilize various carbon sources, the difference in growth conditions *in vitro *compared to *in planta *should be considered. Single carbon sources are tested *in vitro*, whereas *in planta *many different sources are present. For the moment, it is not clear whether the carbon sources differentially used by *Microdochium *spp. *in vitro *are available at contrasting levels in roots or whether they have physiological importance for the fungi. Furthermore, competition with other endophytes for carbon sources may also influence their occurrences in the field. Thus, the challenging task remains to prove that differential utilization of carbon sources *in vitro *contributes to the coexistence of endophytes *in planta*.

Interactions between species implied by positive or negative co-occurrence was the third factor examined with respect to the differential colonization of the roots of common reed by *Microdochium *spp. Although spatial niche partitioning between *M. bolleyi *and *M. phragmitis *was significant, it was not perfect. Since none of the comparisons assessed by Fisher's Exact test exhibited any negative co-occurrence, a direct antagonism between these two species is unlikely. Moreover, in 8.4% of the samples both species were detected which may suggest "true" coexistence. Otherwise, reduced competition for space or carbon (or other essential compounds and ions) may explain this finding. This could occur if colony sizes were much smaller than sample sizes or if the two species used different resources. However, the two *Microdochium *species constitute only a small part of the entire fungal community colonizing common reed. Thus, antagonism or synergism might be indicated when considering additional fungi. This is already seen when including the data available for three more species. Whereas the EcoSim analysis suggests an overall signature of negative co-occurrence, Fisher's Exact test indicates negative and positive co-occurrences for certain species pairings. It is noteworthy that none of the three additional species exhibited negative co-occurrence with *M. bolleyi *and *M. phragmitis *in the total data set. Instead, *M. bolleyi *generally co-occurred significantly more frequently with Ms7Mb4 and Ms43Mb21 than expected by chance. Such a positive co-occurrence may appear when the conditions that are conducive for one species are also favorable for another species. Alternatively, positive co-occurrence may result from synergism. On the other hand, there existed an overall negative co-occurrence between *Stagonospora *sp. and Ms7Mb4, significantly preferring leaves [17] and roots [15], respectively. This could have resulted from strongly contrasting niche preferences, severe competition for the same substrates or from the secretion of toxins (antagonism). Our results suggest that it is rather unlikely that antagonism by any of the other three fungi is responsible for the differential colonization of roots by *Microdochium *spp. Since the fungal community on common reed is larger than addressed here, we cannot rule out that other endophytes may exert such influences.

## Conclusions

This study supports the concept that niche partitioning allows for differential colonization of common reed by the fungal species investigated. Therefore, a purely neutral model is unlikely to explain the assembly of the mycoflora of common reed. Nonetheless, it remains to be shown to what extent stochastic factors could also contribute to variations in the composition, distribution and diversity of this fungal community.

## Authors' contributions

ME collected samples, performed growth rate and nested-PCR assays, statistical data analyses, and contributed to the manuscript. KN collected samples, generated DNA sequences, and conducted the BIOLOG experiments. KWM was an advisor of the work and contributed to the manuscript. SGRW conceived and coordinated the project, contributed to statistical analyses, and wrote the manuscript. All authors read and approved the final manuscript.

## Supplementary Material

Additional file 1 **Details of isolates studied**. This file provides a list of 21 *Microdochium *isolates used in this study, including accession numbers of ITS sequences and information about their origins. Click here for file

Additional file 2 **Specificity of nested-PCR assays targeting *Microdochium *spp**. This file documents the specificity of the assays employed. A) First PCR step using primers ITS1F and ITS4. M = 100 bp size standard, water: no template DNA included, *P. australis*: genomic DNA of axenically grown reed plants, genomic DNAs from fungal isolates 4/97-9 (*Humicola *sp.), 6/97-38 (*Chaetomium *sp.), 6/97-54 (*Fusarium *sp.), A4 (*Fusarium *sp.), 5/97-16 (*Microdochium phragmitis*), 5/97-54 (*M. bolleyi*), 5/97-49 (*M. bolleyi*), 5/97-66 (*M. phragmitis*), respectively. B) and C) Second PCR steps using primers 5/97-16/ITS.F2 and 5/97-16/ITS.R2, and 5/97-54/ITS.F2 and 5/97-54/ITS.R2, respectively, and the products of the first PCR step as templates. Click here for file

Additional file 3 **Utilization of carbon sources**. This file documents relative growth of *Microdochium *isolates on 95 different carbon sources on BIOLOG SF-N2 microtiter plates. Click here for file

Additional file 4 **Pair-wise analysis of spatial niche differentiation**. This file includes P-values from binomial distribution tests for pair-wise analysis of occurrence between five fungal species from reed with respect to space and time. This data set was used to create Figure 5A and 5B. Click here for file

Additional file 5 **Pair-wise analysis of co-occurrence**. This file includes P-values from Fisher's Exact tests for pair-wise analysis of co-occurrence between five fungal species from reed with respect to space and time. Click here for file
