# Supplementary material for: Niche differentiation of two sympatric species of Microdochium colonizing the roots of common reed
Source: BMC Microbiol. 2011 Oct 27;11:242. doi: 10.1186/1471-2180-11-242 (PMC3216463; doi:10.1186/1471-2180-11-242)
Supplement: Additional file 1 — Details of isolates studied. This file provides a list of 21 Microdochium isolates used in this study, including accession numbers of ITS sequences and information about their origins. [file 1471-2180-11-242-S1.PDF]

Additional file 1. Accession numbers of ITS sequences and collection details of the isolates studied

isolate	species	accession	isolated from		habitat	location	ref
			species	organ	type		
A7	*M. bolleyi*	AJ279454	*P. australis*	root	dry	Mainau	#
A12	*M. phragmitis*	AJ279458	*P. australis*	root	dry	Reichenau	#
4/97-7	*M. bolleyi*	AJ279485	*P. australis*	root	flooded	Reichenau	#
4/97-39	*M. phragmitis*	AM502255	*P. australis*	stem	dry	Mainau	+
4/97-103	*M. bolleyi*	AJ279489	*P. australis*	root	flooded	Mainau	#
5/97-16	*M. phragmitis*	AJ279481	*P. australis*	root	dry	Mainau	#
5/97-25	*M. phragmitis*	AM502256	*P. australis*	stem	flooded	Mainau	+
5/97-30	*M. phragmitis*	AM502257	*P. australis*	root	flooded	Mainau	+
5/97-31	*M. phragmitis*	AM502258	*P. australis*	root	flooded	Reichenau	+
5/97-37	*M. phragmitis*	AM502259	*P. australis*	stem	flooded	Mainau	+
5/97-48	*M. bolleyi*	AJ279477	*P. australis*	root	flooded	Reichenau	#
5/97-49	*M. bolleyi*	AM502261	*P. australis*	root	flooded	Reichenau	+
5/97-54	*M. bolleyi*	AJ279475	*P. australis*	root	dry	Mainau	#
5/97-56	*M. bolleyi*	AM502262	*P. australis*	root	dry	Mainau	+
5/97-66	*M. phragmitis*	AM502263	*P. australis*	stem	dry	Mainau	+
6/97-20	*M. phragmitis*	AJ279470	*P. australis*	root	flooded	Mainau	#
CBS137.64	*M. bolleyi*	AM502264	*Secale cereale*	rhizo-sphere	n.d.	Netherlands	*
CBS172.63	*M. bolleyi*	AM502265	n.a.	n.a.	soil of wheat field	Kiel-Kitzeberg, Germany	*
CBS110.94	*M. nivale*	AM502266	*Triticum aestivum*	stem base	n.d.	Italy	*
CBS320.78	*M. nivale*	AM502260	*Triticum aestivum*	n.d.	n.d.	Germany	*
CBS285.71	*M. phragmitis*	AJ279449	*P. australis*	Teleuto-sorus of *Puccinia* sp.	n.d.	Bialowiesza national parc, Poland	# *

+ The ITS sequence was established during this study, the isolate originates from Wirsel et al., 2001. Reichenau and Mainau refer to the two locations at Lake Constance that were sampled. # Both, the ITS sequence and the strain were described in Wirsel et al., 2001.

* Isolation details as on the CBS listing. n.a. not applicable; n.d. not detailed.
