# Supplementary figures and images for: Niche differentiation of two sympatric species of Microdochium colonizing the roots of common reed
Source: BMC Microbiol. 2011 Oct 27;11:242. doi: 10.1186/1471-2180-11-242 (PMC3216463; doi:10.1186/1471-2180-11-242)

## Slide 1
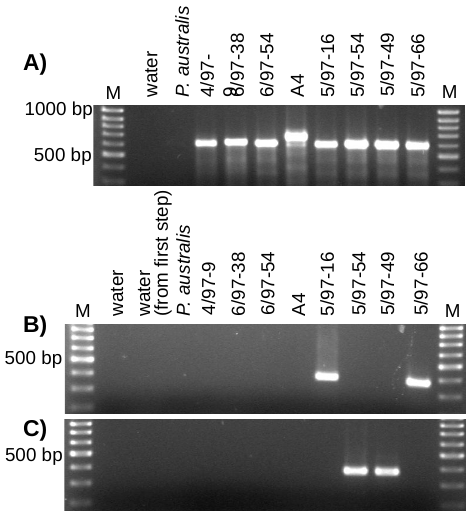

P. australis
A)
5/97-49
5/97-54
6/97-54
5/97-66
5/97-16
6/97-38
4/97-9
water
A4
M
M
1000 bp
500 bp
water
(from first step)
P. australis
6/97-54
5/97-66
5/97-49
5/97-16
5/97-54
4/97-9
6/97-38
water
A4
M
M
B)
500 bp
C)
500 bp

Supplement: Additional file 2 — Specificity of nested-PCR assays targeting Microdochium spp. This file documents the specificity of the assays employed. A) First PCR step using primers ITS1F and ITS4. M = 100 bp size standard, water: no template DNA included, P. australis: genomic DNA of axenically grown reed plants, genomic DNAs from fungal isolates 4/97-9 (Humicola sp.), 6/97-38 (Chaetomium sp.), 6/97-54 (Fusarium sp.), A4 (Fusarium sp.), 5/97-16 (Microdochium phragmitis), 5/97-54 (M. bolleyi), 5/97-49 (M. bolleyi), 5/97-66 (M. phragmitis), respectively. B) and C) Second PCR steps using primers 5/97-16/ITS.F2 and 5/97-16/ITS.R2, and 5/97-54/ITS.F2 and 5/97-54/ITS.R2, respectively, and the products of the first PCR step as templates. [file 1471-2180-11-242-S2.PPT]
