# Supplementary material for: Niche differentiation of two sympatric species of Microdochium colonizing the roots of common reed
Source: BMC Microbiol. 2011 Oct 27;11:242. doi: 10.1186/1471-2180-11-242 (PMC3216463; doi:10.1186/1471-2180-11-242)
Supplement: Additional file 3 — Utilization of carbon sources. This file documents relative growth of Microdochium isolates on 95 different carbon sources on BIOLOG SF-N2 microtiter plates. [file 1471-2180-11-242-S3.PDF]

Additional file 3. Utilisation of carbon sources

	Microdochium bolleyi							*Microdochium phragmitis*						
carbon source	A7	4/97-7	5/97-48	5/97-49	5/97-54	mean	SD	4/97-39	5/97-16	5/97-30	6/97-20	mean	SD	*P*
Control	0.0	0.0	0.0	0.0	0.0	0.0	0.0	0.0	0.0	0.0	0.0	0.0	0.0	n.a.
α-Cyclodextrin (*)	16.9	53.8	55.1	38.0	24.2	37.6	17.1	28.7	4.0	17.3	1.6	12.9	12.6	0.048
Dextrin	84.9	88.3	80.3	88.2	90.0	86.3	3.8	79.3	94.9	73.7	91.5	84.8	10.0	0.769
Glycogen	67.2	55.2	63.8	60.2	55.9	60.5	5.1	66.6	60.1	71.1	62.8	65.1	4.8	0.206
Tween 40 (*)	74.5	92.6	100.0	83.4	70.2	84.1	12.4	55.9	53.6	71.6	72.1	63.3	9.9	0.029
Tween 80	100.0	100.0	84.8	100.0	83.6	93.7	8.7	72.8	61.2	95.0	85.1	78.5	14.7	0.093
N-Acetyl-D-Galactosamine (*)	23.8	31.1	36.0	29.5	24.6	29.0	5.0	17.8	10.4	26.3	2.7	14.3	10.1	0.024
N-Acetyl-D-Glucosamine (+)	67.1	55.0	54.9	55.4	63.2	59.1	5.7	69.2	66.1	67.3	64.9	66.9	1.8	0.035
Adonitol	42.0	48.6	48.0	52.3	37.7	45.7	5.8	64.1	49.9	70.5	43.3	57.0	12.5	0.114
L-Arabinose	51.3	73.7	66.8	58.3	66.3	63.3	8.6	84.4	40.4	47.8	61.5	58.5	19.3	0.634
D-Arabitol (+)	60.0	45.8	53.0	49.0	42.5	50.1	6.8	71.5	52.8	63.0	68.3	63.9	8.2	0.027
D-Cellobiose	65.9	39.8	40.1	87.6	49.9	56.7	20.3	87.3	62.1	51.6	88.6	72.4	18.5	0.269
i-Erythritol (+)	47.0	44.0	52.0	35.2	53.3	46.3	7.2	78.6	42.5	67.3	84.9	68.3	18.7	0.045
D-Fructose (+)	91.6	81.7	79.5	81.5	78.8	82.6	5.2	93.9	95.5	83.3	100.0	93.2	7.1	0.035
L-Fucose	55.3	57.0	47.2	42.6	27.5	45.9	11.8	26.1	9.1	48.2	14.7	24.5	17.3	0.063
D-Galactose	62.8	64.9	67.1	63.8	57.9	63.3	3.4	72.4	65.8	51.7	70.2	65.0	9.3	0.710
Gentiobiose	87.4	88.1	84.3	84.5	96.3	88.1	4.9	77.4	84.2	83.5	85.4	82.7	3.6	0.104
α-D-Glucose	81.1	93.3	90.4	89.0	100.0	90.8	6.9	88.2	92.7	91.3	95.8	92.0	3.2	0.748
m-Inositol	22.0	42.9	61.2	51.3	40.6	43.6	14.6	35.3	22.8	23.0	23.0	26.0	6.2	0.060
α-D-Lactose	21.7	58.2	64.1	54.9	56.3	51.1	16.8	72.4	52.4	17.4	48.9	47.8	22.7	0.810
Lactulose (+)	53.4	41.5	42.2	42.6	29.3	41.8	8.5	73.6	53.9	64.7	50.2	60.6	10.6	0.021
Maltose	80.0	78.6	81.3	77.3	84.8	80.4	2.9	74.5	83.5	80.0	89.1	81.8	6.1	0.662
D-Mannitol (+)	74.2	69.8	71.6	71.5	80.9	73.6	4.4	78.7	81.4	77.5	79.0	79.2	1.6	0.048
D-Mannose	89.2	66.8	64.6	71.0	77.0	73.7	9.9	82.5	100.0	87.8	78.9	87.3	9.2	0.073
D-Melibiose (+)	61.3	46.6	31.5	21.8	22.6	36.7	17.0	66.6	63.0	75.6	78.9	71.0	7.5	0.007
β-Methyl-D-Glucoside	87.8	59.0	71.7	67.3	81.5	73.5	11.4	78.9	74.4	76.9	87.7	79.5	5.8	0.375
D-Psicose (*)	20.7	39.7	42.0	46.7	27.3	35.3	10.8	18.4	9.9	15.9	0.0	11.1	8.2	0.008
D-Raffinose (+)	70.6	50.2	60.8	46.6	57.9	57.2	9.4	80.2	75.3	90.8	84.4	82.7	6.6	0.003
L-Rhamnose	47.8	47.5	65.9	62.9	55.0	55.8	8.5	65.7	46.6	38.7	61.8	53.2	12.7	0.720
D-Sorbitol	29.0	45.0	54.6	49.4	52.2	46.0	10.2	48.2	30.6	36.0	39.7	38.6	7.4	0.266
Sucrose	94.7	79.6	80.1	74.0	84.4	82.6	7.8	80.0	82.5	68.8	90.2	80.4	8.9	0.707
D-Trehalose	85.4	68.8	81.5	76.9	82.6	79.0	6.5	77.0	91.8	87.1	81.5	84.4	6.5	0.260
Turanose	90.5	87.5	99.1	90.1	88.1	91.0	4.7	100.0	91.9	100.0	91.5	95.9	4.8	0.172
Xylitol	50.6	46.1	48.1	53.2	33.3	46.3	7.7	31.8	36.6	48.3	40.8	39.4	7.0	0.210
Pyruvic Acid Methyl Ester	53.1	44.1	47.3	45.6	34.6	44.9	6.7	50.5	25.9	38.9	44.2	39.9	10.4	0.402
Succinic Acid Mono-Methyl Ester	36.4	29.8	38.6	33.8	25.7	32.8	5.2	51.5	24.7	56.0	55.3	46.9	14.9	0.087
Acetic Acid	21.2	10.9	11.1	15.4	16.1	14.9	4.2	14.7	13.8	10.4	11.8	12.6	1.9	0.357
cis-Aconitic Acid (*)	33.2	45.5	34.2	43.3	40.7	39.4	5.5	30.3	26.6	34.4	31.2	30.6	3.2	0.026
Citric Acid	68.4	49.7	59.4	54.6	46.0	55.6	8.7	53.0	48.8	87.5	39.0	57.1	21.1	0.891
Formic Acid	0.0	3.5	7.3	13.2	5.3	5.9	4.9	6.7	2.0	0.9	6.3	4.0	3.0	0.522
D-Galactonic Acid Lactone	1.5	33.6	39.7	26.9	2.7	20.9	17.8	20.4	5.7	0.0	2.0	7.0	9.2	0.203
D-Galacturonic Acid	42.7	25.6	40.0	41.7	29.1	35.8	7.9	48.7	37.6	52.2	40.2	44.7	6.9	0.121
D-Gluconic Acid	49.3	29.7	35.0	20.5	23.6	31.6	11.3	25.4	19.0	52.6	21.8	29.7	15.5	0.836
D-Glucosaminic Acid	0.0	18.0	18.7	12.6	4.0	10.6	8.4	9.4	0.2	0.0	0.2	2.4	4.6	0.125
D-Glucuronic Acid	31.0	31.3	43.1	39.0	32.6	35.4	5.4	37.4	31.7	30.1	32.9	33.0	3.1	0.461
α-Hydroxybutyric Acid	0.0	17.4	12.8	7.9	11.1	9.8	6.5	9.8	1.3	0.1	14.2	6.4	6.8	0.459
β-Hydroxybutyric Acid	15.2	47.2	57.5	39.5	18.3	35.6	18.3	39.7	12.0	17.6	39.3	27.2	14.5	0.480
γ-Hydroxybutyric Acid	31.1	33.2	50.9	27.0	1.0	28.6	17.9	35.6	27.2	28.4	26.4	29.4	4.2	0.937
p-Hydroxy-phenlyacetic Acid	35.6	41.6	39.7	36.6	49.5	40.6	5.5	29.0	35.6	45.3	28.2	34.5	7.9	0.214
Itaconic Acid	0.0	0.5	0.0	11.5	3.4	3.1	4.9	2.5	0.6	0.0	2.4	1.4	1.3	0.526
α-Ketobutyric Acid	17.5	15.4	16.7	14.3	5.3	13.8	4.9	19.4	7.5	16.3	7.6	12.7	6.1	0.768
α-Ketoglutaric Acid	25.2	41.6	33.4	34.8	36.4	34.3	5.9	35.0	25.4	16.2	39.3	29.0	10.3	0.362
α-Ketovaleric Acid (+)	1.6	4.6	1.1	5.8	9.2	4.4	3.3	11.2	5.9	9.0	13.6	9.9	3.3	0.041

D,L-Lactic Acid	14.8	28.5	30.6	24.6	23.1	24.3	6.1	24.2	17.5	9.8	24.8	19.1	7.1	0.271
Malonic Acid (*)	42.9	44.5	42.7	39.5	32.8	40.5	4.7	21.7	15.3	11.1	31.9	20.0	9.1	0.003
Propionic Acid	19.0	12.8	31.0	31.6	17.2	22.3	8.5	34.2	19.3	15.6	21.6	22.7	8.1	0.949
Quinic Acid	32.2	42.8	54.0	39.5	57.6	45.2	10.4	44.7	51.4	34.1	52.4	45.6	8.4	0.953
D-Saccharic Acid	37.8	57.0	63.7	47.3	41.1	49.4	10.9	57.1	36.4	50.2	48.2	48.0	8.6	0.840
Sebacic Acid	38.8	39.1	54.9	44.5	30.1	41.5	9.1	56.1	22.5	42.0	46.2	41.7	14.1	0.975
Succinic Acid	12.3	13.9	20.4	19.0	21.4	17.4	4.0	18.8	16.3	14.1	20.4	17.4	2.8	0.992
Bromosuccinic Acid	19.7	20.4	13.1	14.6	18.3	17.2	3.2	22.5	13.2	32.2	16.3	21.0	8.4	0.374
Succinamic Acid	38.0	36.9	45.0	51.0	39.5	42.1	5.9	54.9	40.2	40.4	47.2	45.7	6.9	0.422
Glucuronamide	0.0	8.4	12.9	9.8	2.7	6.8	5.3	4.4	5.2	0.0	0.0	2.4	2.8	0.182
L-Alaninamide	26.2	32.5	49.8	45.3	33.7	37.5	9.8	29.1	12.0	27.6	38.8	26.9	11.1	0.169
D-Alanine	15.4	34.1	46.5	46.1	30.1	34.4	12.9	23.4	6.7	17.7	30.6	19.6	10.1	0.102
L-Alanine	27.1	27.5	48.1	39.2	48.1	38.0	10.4	42.1	57.0	32.3	37.2	42.1	10.7	0.578
L-Alanyl-Glycine	35.0	34.7	34.1	42.6	38.2	36.9	3.6	43.8	29.4	46.0	35.4	38.7	7.7	0.668
L-Asparagine	31.4	19.4	34.2	33.4	45.2	32.8	9.2	35.4	28.1	39.4	30.0	33.2	5.2	0.928
L-Aspartic Acid	40.8	40.4	64.0	55.2	58.5	51.8	10.7	54.8	45.6	45.7	57.2	50.8	6.1	0.876
L-Glutamic Acid	49.3	44.8	53.7	49.9	61.7	51.9	6.4	60.7	44.8	44.9	47.0	49.3	7.6	0.602
Glycyl-L-Aspartic Acid (+)	22.2	21.7	14.1	13.5	23.1	18.9	4.7	32.2	25.9	36.3	32.1	31.6	4.3	0.004
Glycyl-L-Glutamic Acid (+)	34.0	27.8	36.7	20.4	25.3	28.8	6.6	46.9	31.9	48.1	41.8	42.2	7.4	0.024
L-Histidine (*)	12.6	19.2	24.9	27.5	37.8	24.4	9.4	9.2	16.4	8.8	10.7	11.3	3.5	0.035
Hydroxy-L-Proline (*)	24.9	27.9	34.3	38.3	15.3	28.1	8.9	2.0	11.8	23.5	0.0	9.3	10.8	0.024
L-Leucine	0.4	23.9	32.6	29.4	18.6	21.0	12.7	17.6	9.1	7.0	11.5	11.3	4.6	0.193
L-Ornithine	43.5	41.3	40.8	43.8	50.9	44.1	4.0	47.3	33.4	27.3	44.9	38.2	9.5	0.247
L-Phenylalanine	15.3	15.0	27.9	26.3	32.4	23.4	7.8	22.3	13.5	16.5	25.9	19.5	5.6	0.438
L-Proline	38.8	71.2	66.9	69.1	57.2	60.6	13.3	59.0	27.2	45.6	41.1	43.2	13.1	0.090
L-Pyroglutamic Acid	63.3	46.8	57.3	48.7	52.6	53.7	6.7	52.8	46.1	38.4	51.8	47.3	6.6	0.191
D-Serine	14.9	27.7	33.6	25.3	20.6	24.4	7.1	36.2	12.2	17.1	26.3	23.0	10.6	0.813
L-Serine	16.0	29.3	49.8	45.0	42.7	36.5	13.8	39.6	14.2	23.7	42.6	30.0	13.4	0.499
L-Threonine	11.0	25.8	22.9	23.4	21.2	20.9	5.7	29.9	7.2	15.6	20.1	18.2	9.4	0.617
D,L-Carnitine	3.0	20.7	27.6	27.3	8.7	17.5	11.1	10.4	1.3	2.8	4.5	4.7	4.0	0.068
γ-Aminobutyric Acid	51.0	48.8	54.7	67.0	47.5	53.8	7.9	50.4	49.1	51.0	56.9	51.9	3.5	0.662
Urocanic Acid	9.7	9.4	14.4	12.4	19.7	13.1	4.2	6.7	4.0	17.5	4.7	8.2	6.3	0.204
Inosine	57.8	36.8	30.0	31.5	27.4	36.7	12.3	27.4	40.0	35.9	46.0	37.3	7.8	0.931
Uridine	10.9	30.1	28.5	25.7	14.0	21.8	8.8	18.1	8.2	10.8	15.5	13.1	4.5	0.117
Thymidine	1.2	10.7	11.7	10.6	9.5	8.7	4.3	7.5	2.5	0.0	4.5	3.6	3.2	0.087
Phenylethylamine (*)	6.1	20.3	34.3	25.5	29.3	23.1	10.8	6.3	0.0	0.0	5.0	2.8	3.3	0.009
Putrescine	42.7	38.4	37.6	44.6	46.7	42.0	3.9	42.6	35.8	41.6	41.8	40.4	3.1	0.545
2-Aminoethanol	15.5	35.7	55.6	30.9	5.3	28.6	19.3	57.8	28.6	23.9	50.2	40.1	16.4	0.375
2,3-Butanediol	3.7	4.6	11.0	7.7	10.7	7.5	3.4	7.7	2.2	3.8	3.0	4.2	2.4	0.141
Glycerol	11.0	32.3	39.9	40.6	22.1	29.2	12.6	26.5	8.2	16.7	20.0	17.8	7.6	0.160
D,L,α-Glycerol Phosphate	4.2	5.8	13.4	9.4	11.4	8.8	3.8	7.6	2.0	2.6	3.0	3.8	2.6	0.061
α-D-Glucose-1-Phosphate (*)	10.2	9.9	19.4	15.3	13.7	13.7	3.9	9.8	4.7	7.5	3.3	6.3	2.9	0.017
D-Glucose-6-Phosphate	8.9	4.8	12.2	8.4	14.9	9.8	3.9	7.2	4.0	8.5	2.1	5.4	2.9	0.103

Growth on BIOLOG SF-N2 microtiter plates was recorded after 10 d as the percentage of the maximal value obtained for any substrate. The first well contained no carbon source and served to adjust the baseline. The last column gives the results of a t-test assessing the variance between the two species. Characters behind the carbon source indicate significant better growth of either *M. bolleyi* (*) or *M. phragmites* (+).
