# Supplementary material for: Niche differentiation of two sympatric species of Microdochium colonizing the roots of common reed
Source: BMC Microbiol. 2011 Oct 27;11:242. doi: 10.1186/1471-2180-11-242 (PMC3216463; doi:10.1186/1471-2180-11-242)
Supplement: Additional file 4 — Pair-wise analysis of spatial niche differentiation. This file includes P-values from binomial distribution tests for pair-wise analysis of occurrence between five fungal species from reed with respect to space and time. This data set was used to create Figure 5A and 5B. [file 1471-2180-11-242-S4.PDF]

Additional file 4. Pairwise analysis of spatial niche differentiation

		M. bolleyi	***Stagonospora* sp.**	**Ms7Mb4**	**Ms43Mb21**
**total**	*M. phragmitis*	0.1766	0.2615	0.0214	0.0128
	M. bolleyi		1.0000	0.3620	**0.000011**
	Stagonospora sp.			0.4153	**0.00044**
	Ms7Mb4				**0.0000001**
**April**	*M. phragmitis*	1.2461	0.0018	0.1435	0.3018
	M. bolleyi		**0.0005**	0.0654	0.3018
	Stagonospora sp.			**0.0000038**	0.0391
	Ms7Mb4				0.0169
**June**	*M. phragmitis*	0.6072	0.0215	0.8036	0.0010
	M. bolleyi		0.0074	0.3018	**0.0001**
	Stagonospora sp.			0.1094	0.2500
	Ms7Mb4				0.0039
**July**	*M. phragmitis*	0.1797	0.7266	0.0386	0.6875
	M. bolleyi		0.5488	0.5078	0.4531
	Stagonospora sp.			0.1796	1.2461
	Ms7Mb4				0.0703
**August**	*M. phragmitis*	1.2256	0.0025	0.0010	1.5760
	M. bolleyi		0.0025	0.00235	0.6875
	Stagonospora sp.			0.7283	0.0161
	Ms7Mb4				0.0074
**September**	*M. phragmitis*	0.0963	**0.0001**	1.0000	0.7744
	M. bolleyi		0.0755	0.0923	0.1460
	Stagonospora sp.			0.0023	0.0037
	Ms7Mb4				1.0000
**November**	*M. phragmitis*	0.6072	0.4545	0.3018	0.0018
	M. bolleyi		1.0000	0.7744	0.0039
	Stagonospora sp.			1.0000	0.0215
	Ms7Mb4				0.0391
**leaf**	*M. phragmitis*	1.2256	**0.000034**	0.0156	0.0703
	M. bolleyi		**0.0000015**	0.0156	0.0703
	Stagonospora sp.			**0.0000000019**	**0.0000000037**
	Ms7Mb4				1.0000
**stem**	*M. phragmitis*	1.1855	0.0433	1.0000	0.0225
	M. bolleyi		0.0708	1.0000	0.0352
	Stagonospora sp.			0.1102	**0.00018**
	Ms7Mb4				0.0129
**root**	*M. phragmitis*	0.2430	0.0037	**0.00051**	0.2559
	M. bolleyi		**0.000019**	0.0192	0.0037
	Stagonospora sp.			**0.00000000078**	0.1153
	Ms7Mb4				**0.00000013**
**rhizome**	*M. phragmitis*	0.2668	0.1460	0.2100	1.0000
	M. bolleyi		0.0127	0.8388	0.3877
	Stagonospora sp.			0.0044	0.0923
	Ms7Mb4				0.2863
**dry habitat**	*M. phragmitis*	**0.0002**	0.1263	0.0043	0.4869
	M. bolleyi		0.1818	0.6177	0.0029
	Stagonospora sp.			0.3663	0.4709
	Ms7Mb4				0.0288

**flooded habitat**	*M. phragmitis*	0.1641	1.0000	0.760	**0.000014**
	M. bolleyi		0.0989	0.0596	**0.0023**
	Stagonospora sp.			0.8991	**0.000015**
	Ms7Mb4				**0.00000007**
**leaf-dry**	*M. phragmitis*	1.0000	0.0034	0.5000	1.0000
	M. bolleyi		0.0018	1.0000	1.5000
	Stagonospora sp.			**0.0002**	**0.0005**
	Ms7Mb4				1.0000
**stem-dry**	*M. phragmitis*	1.0000	0.0352	0.2891	0.3750
	M. bolleyi		0.0963	0.5078	0.2188
	Stagonospora sp.			0.3593	0.0042
	Ms7Mb4				0.0156
**root-dry**	*M. phragmitis*	**0.00004**	0.5078	0.0013	0.2101
	M. bolleyi		**0.000019**	0.3438	0.0042
	Stagonospora sp.			**0.00008**	0.0117
	Ms7Mb4				0.0386
**rhizome-dry**	*M. phragmitis*	0.1797	0.0313	0.5811	0.5078
	M. bolleyi		0.0010	0.8036	0.7539
	Stagonospora sp.			0.0039	0.0039
	Ms7Mb4				1.1964
**leaf-flooded**	*M. phragmitis*	1.0000	0.0075	0.0625	0.0625
	M. bolleyi		0.0010	0.0313	0.0313
	Stagonospora sp.			**0.000015**	**0.000015**
	Ms7Mb4				2.0000
**stem-flooded**	*M. phragmitis*	1.0000	0.7539	0.5488	0.0703
	M. bolleyi		0.5811	0.7539	0.1797
	Stagonospora sp.			0.2668	0.0386
	Ms7Mb4				0.4531
**root-flooded**	*M. phragmitis*	0.0213	0.0044	0.1435	0.0043
	M. bolleyi		0.4531	**0.000015**	0.3877
	Stagonospora sp.			**0.000011**	1.0000
	Ms7Mb4				**0.000001**
**rhizome-flooded**	*M. phragmitis*	1.3750	1.3125	0.3438	0.6250
	M. bolleyi		1.3125	0.2891	0.5000
	Stagonospora sp.			0.3438	0.6250
	Ms7Mb4				0.0313

Numbers are *P*-values from binomial distribution tests ($P < 0.05$). Pink underlay indicates a significant higher incidence of the fungus at the left, and respectively an yellow underlay of the fungus at the top row. Bold types indicate differences remaining significant after Bonferroni correction (total $P = 0.005$, season $P = 0.000833$, organ $P = 0.00125$, habitat $P = 0.0025$, organ-habitat $P = 0.000625$).
