# Supplementary material for: Niche differentiation of two sympatric species of Microdochium colonizing the roots of common reed
Source: BMC Microbiol. 2011 Oct 27;11:242. doi: 10.1186/1471-2180-11-242 (PMC3216463; doi:10.1186/1471-2180-11-242)
Supplement: Additional file 5 — Pair-wise analysis of co-occurrence. This file includes P-values from Fisher's Exact tests for pair-wise analysis of co-occurrence between five fungal species from reed with respect to space and time. [file 1471-2180-11-242-S5.PDF]

Additional file 5. Pairwise analysis of co-occurrence

		M. bolleyi	*Stagonospora* sp.	Ms7Mb4	Ms43Mb21
**total**	*M. phragmitis*	0.0361	0.4898	0.0305	1.0000
	M. bolleyi		0.2612	**0.0002**	**0.0002**
	Stagonospora sp.			**0.0017**	0.0833
	Ms7Mb4				**0.0003**
**April**	*M. phragmitis*	0.0298	1.0000	0.7314	1.0000
	M. bolleyi		0.3611	0.0140	1.0000
	Stagonospora sp.			1.0000	1.0000
	Ms7Mb4				0.4217
**June**	*M. phragmitis*	0.2579	0.1272	1.0000	n.co.
	M. bolleyi		1.0000	0.4162	n.co.
	Stagonospora sp.			0.4716	n.co.
	Ms7Mb4				n.co.
**July**	*M. phragmitis*	0.5082	1.0000	1.0000	0.3452
	M. bolleyi		1.0000	0.0276	0.0491
	Stagonospora sp.			1.0000	1.0000
	Ms7Mb4				0.0169
**August**	*M. phragmitis*	1.0000	0.4158	0.0158	1.0000
	M. bolleyi		0.4158	0.1115	0.0013
	Stagonospora sp.			0.0095	0.0238
	Ms7Mb4				0.0238
**September**	*M. phragmitis*	0.6561	0.6662	0.2061	1.0000
	M. bolleyi		0.7516	0.1711	0.0365
	Stagonospora sp.			0.0971	0.2448
	Ms7Mb4				0.0100
**November**	*M. phragmitis*	1.0000	1.0000	1.0000	1.0000
	M. bolleyi		1.0000	0.6808	0.1109
	Stagonospora sp.			0.2100	0.5343
	Ms7Mb4				0.4899
**leaf**	*M. phragmitis*	1.0000	0.6860	n.co.	1.0000
	M. bolleyi		0.1167	n.co.	1.0000
	Stagonospora sp.			n.co.	1.0000
	Ms7Mb4				n.co.
**stem**	*M. phragmitis*	0.2669	0.5230	0.4496	0.1712
	M. bolleyi		0.3454	0.4496	1.0000
	Stagonospora sp.			0.2274	0.2880
	Ms7Mb4				0.1951
**root**	*M. phragmitis*	0.6123	1.0000	0.7899	0.0104
	M. bolleyi		1.0000	0.1798	0.2600
	Stagonospora sp.			0.7115	0.6844
	Ms7Mb4				0.0693
**rhizome**	*M. phragmitis*	0.0177	1.0000	0.4326	0.1345
	M. bolleyi		1.0000	1.0000	0.0046
	Stagonospora sp.			0.5692	1.0000
	Ms7Mb4				1.0000
**dry habitat**	*M. phragmitis*	0.0037	0.7793	0.1882	0.2317
	M. bolleyi		**0.0008**	**0.0002**	**0.0002**
	Stagonospora sp.			0.0446	0.3097
	Ms7Mb4				0.0068

**flooded habitat**	*M. phragmitis*	0.4506	0.6552	0.0834	0.6732
	M. bolleyi		0.0415	0.2116	1.0000
	Stagonospora sp.			0.0281	0.1888
	Ms7Mb4				0.0228
**leaf-dry**	*M. phragmitis*	1.0000	1.0000	n.co.	1.0000
	M. bolleyi		1.0000	n.co.	1.0000
	Stagonospora sp.			n.co.	0.4815
	Ms7Mb4				n.co.
**stem-dry**	*M. phragmitis*	0.0313	1.0000	0.1110	0.2841
	M. bolleyi		0.2085	0.3092	0.3352
	Stagonospora sp.			0.2409	0.4962
	Ms7Mb4				0.0682
**root-dry**	*M. phragmitis*	0.6518	1.0000	1.0000	0.6756
	M. bolleyi		0.2412	0.1057	0.4311
	Stagonospora sp.			1.0000	0.2757
	Ms7Mb4				0.0672
**rhizome-dry**	*M. phragmitis*	0.1476	n.co.	0.6482	0.3140
	M. bolleyi		n.co.	0.4414	0.2134
	Stagonospora sp.			n.co.	n.co.
	Ms7Mb4				0.383
**leaf-flooded**	*M. phragmitis*	1.0000	0.3261	n.co.	n.co.
	M. bolleyi		0.0570	n.co.	n.co.
	Stagonospora sp.			n.co.	n.co.
	Ms7Mb4				n.co.
**stem-flooded**	*M. phragmitis*	0.6518	0.2054	1.0000	0.4318
	M. bolleyi		1.0000	1.0000	1.0000
	Stagonospora sp.			1.0000	1.0000
	Ms7Mb4				1.0000
**root-flooded**	*M. phragmitis*	0.6992	0.6390	0.6992	0.0334
	M. bolleyi		0.0784	0.1516	0.5503
	Stagonospora sp.			1.0000	1.0000
	Ms7Mb4				0.5503
**rhizome-flooded**	*M. phragmitis*	0.2559	1.0000	1.0000	1.0000
	M. bolleyi		1.0000	0.5235	0.0909
	Stagonospora sp.			1.0000	1.0000
	Ms7Mb4				0.2121

Numbers are *P*-values from Fisher´s Exact test ($P < 0.05$). Green underlay denotes significantly more, Red underlay denotes significantly less co-occurrence than expected by chance. n.co.: no co-occurrence observed. Bold types indicate differences remaining significant after Bonferroni correction (total $P = 0.005$, season $P = 0.000833$, organ $P = 0.00125$, habitat $P = 0.0025$, organ-habitat $P = 0,000625$).
